# 532 nm green-light laser vaporization of upper tract urothelial carcinoma

**DOI:** 10.1186/s12894-020-00744-w

**Published:** 2020-10-28

**Authors:** F. Dursun, M. M. Pan, M. Morgan, R. R. Gonzalez, R. Satkunasivam

**Affiliations:** 1 Department of Urology, Houston Methodist Hospital, 6560 Fannin Street, Suite 2100, Houston, TX 77030 USA; 2 Scott Department of Urology, Baylor College of Medicine, Houston, TX USA

**Keywords:** Upper tract urothelial carcinoma, Endoscopic management, Laser ablation, Greenlight, KTP

## Abstract

**Background:**

Endoscopic management of low risk upper tract urothelial carcinoma (UTUC) may be considered in select clinical scenarios, which allows sparing the morbidity of radical nephroureterectomy while achieving acceptable oncological outcomes and preservation of kidney function. Herein, we present a case with UTUC in a solitary kidney managed with 532 nm laser vaporization through a percutaneous approach.

**Case presentation:**

The patient in this video (Additional file 1) is an 85-year-old woman who presented with a bulky tumor in the collecting system of a congenital solitary left kidney, which was a biopsy proven low grade urothelial carcinoma. Prior to the procedure, a lower pole percutaneous nephrostomy tube was successfully placed under sedation by Interventional Radiology. The procedure was done in a prone split leg position. The mass, which was predominantly localized to the renal pelvis was efficiently vaporized with the 532 nm laser in a systematic manner with continuous irrigation of normal saline through the cystoscope. The patient was discharged home on postoperative day 2 with the nephroureterostomy catheter open to drainage. This catheter was subsequently clamped and removed two weeks later without complications. Follow up uretroscopy showed excellent treatment response and the patient remains well without complications.

**Conclusion:**

This case report details the potential utility of 532 nm laser vaporization of UTUC, however, ongoing studies are required to demonstrate peri-operative safety and durable oncologic efficacy.

## Background

Upper tract urothelial carcinomas (UTUC) are uncommon and account for only 5–10% of urothelial carcinoma with a peak incidence in individuals aged 70–90 years [1, 2]. Nephroureterectomy with bladder-cuff removal remains the gold standard for the management of UTUC [3]. Endoscopic management may be considered in select clinical scenarios. Kidney-sparing surgery (KSS) for low risk UTUC is an option that allows sparing the morbidity of radical nephroureterectomy (RNU) while achieving acceptable oncological outcomes and preservation of kidney function. In low grade cancers it is the preferred approach, with survival being similar after KSS versus RNU [4]. In addition, it can also be considered in select patients with significant renal insufficiency or a solitary kidney.

At present, the 532 nm lithium triborate laser, also known as green-light laser, is a popular and effective surgical method for treating benign prostatic hyperplasia given its tissue affinity for hemoglobin and reduced risk of bleeding complications compared to standard resection [5]. En-bloc vapoenucleation with 532 nm laser is also reported as a feasible, safe and effective option for the management of select non-muscle invasive bladder cancer patients [6]. Given the established properties of the 532 nm laser, we sought to utilize this approach in the percutaneous management of low grade UTUC in a solitary kidney.

## Case presentation

An 85-year-old woman presented with a bulky tumor in the collecting system of a congenital solitary left kidney. Her past medical history includes significant chronic obstructive pulmonary disease with a 2 pack-day smoking history more than 50 years. She has a history of recurrent low-grade non-muscle invasive bladder cancer (NMIBC) for the last 7 years for which she received intravesical BCG treatments. Her last recurrence in the bladder was 3 months prior showing non-invasive low-grade urothelial carcinoma.

**Fig. 1:**
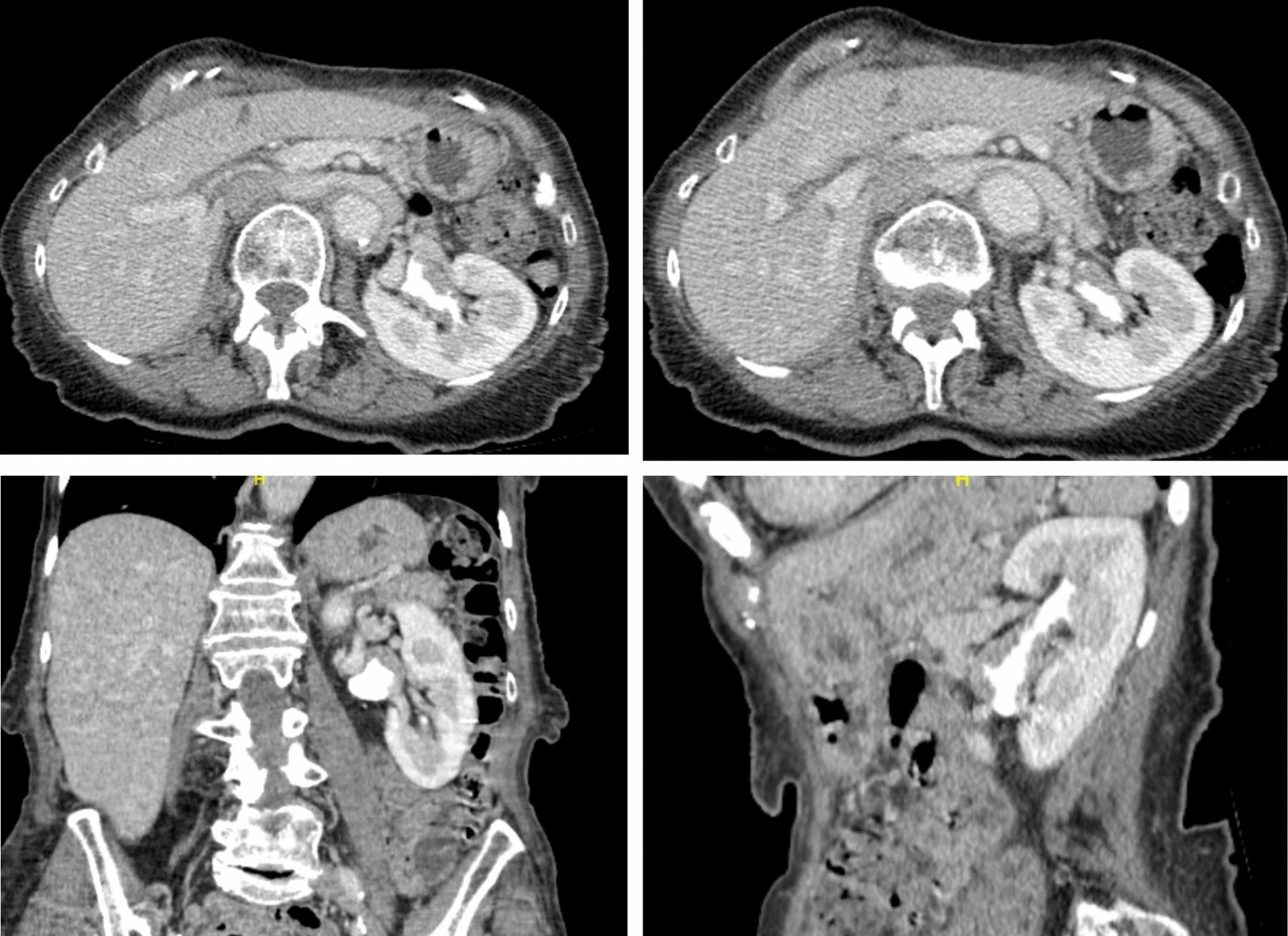
A computerized tomography scan with urogram demonstrated lobulated urothelial thickening at the left renal pelvis, and infundibulum of the upper pole calyx without hydronephrosis

Laboratory tests revealed normal renal function including serum creatinine of 0.69 mg/dl. A computerized tomography (CT) scan with a urographic phase demonstrated lobulated urothelial thickening of the left renal pelvis without hydronephrosis (Fig. 1). CT imaging of the chest did not identify metastatic disease.

The patient underwent flexible ureteroscopy and nephroscopy with ureteral washing and biopsy. A sessile tumor greater than 2 cm was visualized in the renal pelvis towards the upper pole infundibulum. Two adequate specimens were obtained in order to determine the disease grade and were consistent with low grade urothelial carcinoma.

After a detailed discussion with the patient about the risks, benefits, and possible complications of KSS, the patient elected to undergo percutaneous treatment with laser ablation of the UTUC in her solitary kidney. Prior to the procedure, a lower pole percutaneous nephrostomy tube was successfully placed under sedation by Interventional Radiology.

The patient was positioned in prone split leg position. We cystoscopically confirmed that there was no tumor recurrence in the bladder. A guidewire was passed through the nephrostomy tube, ureteroscopically grasped with a nitinol basket and was taken out to achieve through-and-through access. After placing a 12.7 French ureteral access sheath under fluoroscopy, we visualized the renal pelvis again using the ureteroscope and dilated the percutaneous tract with a balloon dilatator under direct vision and placed a 28 French Amplatz sheath, which allowed the introduction of a 24 French continuous-flow cystoscope (Richard Wolf, CITY) with normal saline for irrigation. Importantly, the ureteral access sheath was left to optimize drainage, provide low pressure in the renal pelvis, and limit any tumor seeding in the bladder.

After systematic exploration of the collecting system, the boundaries of the large sessile tumor were visualized, and the renal pelvis mass was vaporized with the 532 nm laser in a systematic manner with continuous irrigation of normal saline through the cystoscope. GreenLight XPS LBO 180W laser console (Boston Scientific, Malborough, MA) was used with an extended application (EA) 600 micron side-fire fiber (Boston Scientific, Malborough, MA). The mass was vaporized using a setting of 40 watts; to coagulate any bleeders, the power was set at 20 watts. Care was taken to stay in near-contact with the mass during vaporization, using visible bubbles as feedback of efficient vaporization and hemostasis of the tumor bed without charring tissue. The hemostasis was achieved effectively in a very short duration at low power. Total energy used during the procedure was 39,515 J. After satisfactory tumor ablation, an 8-French nephroureteral catheter was inserted under fluoroscopic and cystoscopic guidance to maintain adequate urinary drainage.

The patient was discharged home on postoperative day 2 with the nephroureterostomy catheter open to drainage. The catheter was subsequently clamped and removed two weeks later without complications. Follow up included cystoscopy and CT urogram at 3 months. CT urogram demonstrated focal thickening in the upper pole urothelium without obvious recurrence. Ureteroscopy was performed at 6 months which demonstrated minimal residual or recurrent disease (5 mm in upper pole calyx) that was successfully ablated (thulium laser). Excellent treatment response was observed throughout the remaining collecting system. At the same time two small papillary low-grade bladder tumors were resected, followed by intravesical gemcitabine. The patient is now at 8 months of follow up and remains well, with plans for ongoing cross sectional imaging, cystoscopic and ureteroscopic follow up. Throughout the treatment, we did not observe any deleterious consequences to renal function. In the accompanying video (Additional file 1), we present our technique for KTP Green-light laser vaporization of UTUC via a percutaneous approach.

## Discussion and conclusion

Upper tract urothelial carcinoma in a solitary kidney creates challenging decisions for the patient and physician. Radical nephroureterctomy is not without morbidity and the long-term quality of life and morbidity and mortality of dialysis must be considered. Endoscopic approaches are a viable option for the management of UTUC when kidney function preservation is of paramount importance. Percutaneous approaches over retrograde (ureteroscopic) approaches offer advantages with respect to better visualization and flexibility in instrumentation [7]. While laser and resection-based approaches (i.e. bipolar resectoscope) have been described, this case, to our knowledge, represents the first treatment of UTUC with 532 nm laser vaporization.

A variety of lasers have been proven to be useful for the management of superficial and low-grade UTUC without compromising oncologic outcomes or renal function [8]. The high-power 532 nm laser is selectively absorbed by hemoglobin but not by water, leading to rapid tissue vaporization with an optical penetration depth of about 0.8 mm and 2–3 mm zone of coagulation. Although the heat-affected-zone of coagulation is higher than holmium and thulium lasers, selective absorption by hemoglobin provides better coagulation and limited necrosis of the tissue below the vaporized area [9]. This 2 mm rim of coagulation zone under the vaporized area may provide a better safety profile for bleeding complications especially for patients at high risk for clinically significant bleeding [5]. The advantages of 532 nm laser vaporization through a nephrostomy tract includes better visualization as a result of both optimal coagulation and excellent irrigation flow with low intra-pelvic pressures. Limitations include lack of tissue for pathologic diagnosis (hence necessitating pre-operative confirmation of low-grade urothelial carcinoma), the lack of flexibility due to the laser fiber which may limit access to certain tumor locations, and potential perforation intraoperatively or in delayed fashion. Despite the fact that bulky tumors can be handled by this approach, our case highlights the need for ongoing close follow up as repeat ablations may be necessary. This may be particularly true in areas difficult to reach by rigid instruments. The impact on depth of penetration to normal renal tissue and potential renal functional loss as well as other renal complications remains to be carefully followed and therefore must be closely investigated in future studies. Improving outcomes for UTUC may include consideration of novel ablative technologies which have been introduced in the lower urinary tract. Overall, patient selection for endoscopic treatment remains critical and consideration must also be given to multimodal therapy with topical chemotherapeutic strategies [10].

This case report details the potential utility of 532 nm laser vaporization of UTUC, however, ongoing studies are required to demonstrate peri-operative safety and durable oncologic efficacy.

## Supplementary information

**Additional file 1.** Intraoperative video demonstrating 532 nm green-light vaporization of upper tract urothelial carcinoma.

## Data Availability

Not applicable.

## Abbreviations

UTUC: Upper tract urothelial carcinoma
KSS: Kidney-sparing surgery
RNU: Radical nephroureterectomy
NMIBC: Non-muscle invasive bladder cancer
BCG: Bacillus Calmette–Guérin
CT: Computerized tomography
